# A cross-sectional internet-based patient survey of the management strategies for gout

**DOI:** 10.1186/s12906-016-1067-3

**Published:** 2016-03-01

**Authors:** Jasvinder A. Singh, Nipam Shah, N. Lawrence Edwards

**Affiliations:** Medicine Service, Birmingham VA Medical Center, Birmingham, AL USA; Department of Medicine at School of Medicine, and Division of Epidemiology at School of Public Health, University of Alabama, Faculty Office Tower 805B, 510 20th Street S, Birmingham, AL 35294 USA; Department of Orthopedic Surgery, Mayo Clinic College of Medicine, Rochester, MN USA; Department of Medicine, University of Florida, Gainesville, FL USA

**Keywords:** Gout, Cherry extract, Cherry juice, Complementary medicine, Allopurinol, Febuxostat, Internet study, Survey, Natural supplements, Diet modification

## Abstract

**Background:**

Almost half of the patients with gout are not prescribed urate-lowering therapy (ULT) by their health care provider and >50 % use complementary and alternative therapies. Diet modification is popular among gout patients due to known associations of certain foods with gout flares. The interplay of the use of dietary supplements, diet modification, and ULT adherence in gout patients is not known. Despite the recent interest in diet and supplements, there are limited data on their use. Our objective was to assess ULT use and adherence and patient preference for non-pharmacological interventions by patients with gout, using a cross-sectional survey.

**Methods:**

People who self-reported physician-diagnosed gout during their visit to a gout website (http://gouteducation.org) were invited to participate in a brief anonymous cross-sectional Internet survey between 08/11/2014 to 04/14/2015 about the management of their gout. The survey queried ULT prescription, ULT adherence, the use of non-pharmacological interventions (cherry extract, diet modification) and the likelihood of making a lifelong diet modification for gout management.

**Results:**

A total of 499 respondents with a mean age 56.3 years were included; 74 % were males and 74 % were White. Of these, 57 % (285/499) participants were prescribed a ULT for gout, of whom 88 % (251/285) were currently taking ULT. Of those using ULT, 78 % (97/251) reported ULT adherence >80 %. Gender, race, and age were not significantly associated with the likelihood of receiving a ULT prescription or ULT adherence >80 %. Fifty-six percent of patients with gout preferred ULT as a lifelong treatment for gout, 24 % preferred cherry extract and 16 % preferred diet modification (4 % preferred none). Men had significantly lower odds of preferring ULT as the lifelong treatment choice for gout vs. other choices (*p* = 0.03). We found that 38.3 % participants were highly motivated to make a lifelong dietary modification to improve their gout (score of 9–10 on a 0–10 likelihood scale). Older age was significantly associated with high level of willingness to modify diet (*p* = 0.02).

**Conclusion:**

We found that only 57 % of gout patients reported being prescribed ULT. 40 % of gout patients preferred non- pharmacological interventions such as cherry extract and diet modification for gout management. The latter finding requires further investigation.

## Key messages

1. Almost half of gout patients preferred non- pharmacological interventions such as cherry extract and diet modification for long-term gout management.
2. Survey participants were highly motivated to make a lifelong dietary modification to improve their gout.
3. Only half of gout patients participating in Internet study reported being prescribed urate-lowering therapy.

## Background

Gout is the most prevalent type of chronic inflammatory arthritis in adults [1]. Gout is associated with significant pain, disability and a negative impact on quality of life [2–6]. Treatment with urate-lowering therapy (ULT) such as allopurinol, febuxostat, probenecid or pegloticase is key to successful long-term management of gout. The American College of Rheumatology (ACR) and the European League Against Rheumatism (EULAR) guidelines for the management of gout support the use of education and diet alongside pharmacologic interventions [7, 8]. Thus, optimal gout management may require a combination of pharmacological interventions, diet modification and possibly the use of dietary supplements [9, 10].

Many treatment and knowledge gaps exist in the area of gout management. Key treatment gaps include low rates of treatment with ULT and low adherence to ULT medications [11] and the failure to monitor and achieve a target serum urate < 6 mg/dl, an important therapeutic goal associated with better outcomes. A recent Internet study showed that only 55 % of patients with gout were prescribed a ULT by their health care provider [12], a finding similar to that noted in previous studies using large databases [13, 14]. Medication adherence was the lowest for gout among seven chronic conditions studied, with 36 % gout patients with 80 % or higher adherence with medication in the first year of prescription compared to 68 and 65 % of patients with hypothyroidism and diabetes (roughly twice as high as in gout), respectively [11]. Qualitative research has shown that this low adherence is multifactorial [15, 16]. Thus, treatment gaps are common in gout and medication adherence and outcomes are suboptimal.

Gout patients commonly use natural treatments such as cherry extract and cherry juice [10, 12, 17], which may be related to a lower gout flare rate with cherry extract use [10]. In one survey, gout patients prioritized the role of supplements and vitamins in gout as one of the top research questions [18]. Diet plays a significant role in the pathophysiology of gout and therefore diet modification is an important behavioral approach to gout management [19–22]. Dietary modifications recommended for gout include increase in low-fat dairy and Vitamin C, and reduction of total protein, alcohol intake and high-fructose drinks [9, 23]. However, the precise role of dietary supplements (vitamins, minerals, herbs, extracts/concentrates etc.) and dietary modification (prudent or low purine diet) in optimizing gout treatment is yet to be determined.

Despite the recent interest in dietary supplements and diet modification, limited data exist on their use by patients with gout. Specifically, it is unknown how patients view the role of dietary supplements, diet modification, and ULT for the management of their gout. To our knowledge, there are no studies that describe the patient characteristics associated with the use of non-pharmacological interventions for gout. Therefore, we aimed to use a brief focused cross-sectional online survey in patients with gout to assess patient approach to gout management. We assessed whether patient characteristics were associated with the likelihood of receiving ULT prescription, higher ULT adherence, patient preference for gout management strategy (ULT vs. supplements vs. diet modification) and the patient motivation for a lifelong dietary modification for gout management.

## Methods

People visiting the Gout and Uric Acid Education Society’s website (http://gouteducation.org) were invited to participate in a brief anonymous Internet survey on a voluntary basis between 08/11/2014 to 04/14/2015 (survey pop-up turned on for three 1-month intervals). When people visited the website, a pop-up asked them if they had physician-diagnosed gout. Patients reporting physician-diagnosed gout were invited to complete an optional, brief gout survey regarding the use of treatments and dietary supplements for gout. People not interested in the survey could click out of the pop-up.

We collected the following information for respondents with self-reported physician-diagnosed gout: (1) age, gender and race; (2) receipt of physician prescription of urate-lowering medication (ULT; allopurinol or febuxostat; responses were yes/no); (3) number of days the patient forgot to take ULT in the last month (self-reported ULT adherence); (4) the use of cherry extract (made from cherries and usually available as capsules or tablets in health stores in the U.S.) vs. diet modification vs. ULT medication as what individuals thought might be the best life-long strategy for gout; and (5) patients’ likelihood of making a lifelong change to their diet (0 = not at all likely to 10 = extremely likely; 0–10 scale) for better gout management. The Institutional Review Board (i.e., Ethics committee) at the University of Alabama at Birmingham (UAB), Birmingham, Alabama, USA, approved this study.

Descriptive statistics (mean, standard deviation, proportion) were calculated. Dependent variables for the main study outcomes were: (1) ULT prescribed (yes/no); (2) ULT adherence (≤0.80 vs. >0.80); (3) patient preference for best gout management strategy (ULT vs. others); and (4) patient motivation for lifelong dietary modification for gout management (score >8 on 0–10 scale). Independent variables were gender, age (in categories of 21–40, 41–60, 61–80 and >80 years) and race/ethnicity.

We used chi-square test for comparing categories of ULT adherence, treatment choice for gout (pharmacological vs. non-pharmacological) and the likelihood of life-long diet modification by age, gender and race. We used multivariable-adjusted logistic regression model to predict these outcomes. We present odds ratio and 95 % confidence intervals (CI). A p-value <0.05 was considered statistically significant.

Sensitivity analyses were conducted additionally adjusting the main models for patient preference for pharmacological vs. non-pharmacological treatments for gout: (1) ULT prescribed (yes/no); and (2) ULT adherence (≤80 % vs. >80 %). These were examined to assess whether patient preference for non-pharmacological treatments impacts the ULT prescription or ULT adherence.

## Results

Of the 524 survey participants reporting a physician diagnosis of gout, 499 respondents were included in the final analysis (Table 1; 25 people were excluded for un-interpretable responses). The mean age of gout survey responders was 56.3 years (standard deviation, 12.6), 49 % were 41–60 years old, 74 % were males, 74 % were White and 9 % were Asians. Almost half of the female participants were 61–80 years old (46 %) and half of the male participants were 41–60 years old (53 %).

**Table 1:** Characteristics of patients who responded to the Internet survey and self-reported a physician diagnosis of gout (*N* = 499)

Characteristics	N or n/N	%^a^ or mean ± SD
Age, mean ± SD (range)	497	56.3 ± 12.6 (25–93)
Age groups, %		
21–40	61	12.2
41–60	244	48.9
61–80	179	35.9
≥80	13	2.6
Missing	2	0.4
Sex, %		
Male	368	73.7
Female	131	26.3
Race, %		
White/Caucasian	371	74.3
African American	38	7.6
Hispanic or Latino	14	2.8
Asian	45	9.0
American Indian or Alaskan Native	3	0.6
Hawaiian or Pacific Islander	9	1.8
Other	18	3.6
Missing	1	0.2
Patients prescribed Allopurinol/Febuxostat, %	285	57.1
Number of days gout medication missed in past 30 days, mean ± S.D.	251	4.50 ± 8.72
Of those prescribed ULT, n/N (%)		
On ULT	250/285	88
Quit ULT	20/285	7
Taking ULT infrequently	2/285	0.7
Missing/did not respond	13/285	4.5
Days ULT missed in past 30 days, n/N (%)		
0 days (adherence 100 %)	148/251	59
1–5 days (adherence >80 %)	49/251	19.5
6–14 days (adherence, 51–80 %)	26/251	10.3
>15 days (adherence, ≤50 %)	28/251	11.2
Best life-long treatment choice, %		
Cherry extract	122	24.4
Diet modification	79	15.8
ULT	277	55.5
None	21	4.2
Likelihood of making life-long change to diet (0–10), %		
0–8	308	61.7
9–10	191	38.3

^a^All numbers are percent except for age and the number of days gout medication was missed

*ULT* Urate lowering therapy, *SD* standard deviation

**ULT prescription**: 57 % participants reported that they were prescribed a ULT (allopurinol or febuxostat) for gout by their healthcare provider. Gender, age, or race, were not significantly associated with the likelihood of receiving a ULT prescription in multivariable-adjusted analyses (Table 2).

**Table 2:** Multivariate-adjusted odds of ULT prescription, the choice of ULT as the gout treatment (ULT vs. others), adherence to ULT >80 % and likelihood of lifelong diet change as treatment for gout by gender, race and age

Patient characteristics	ULT prescribed^a^	Choice of ULT as the treatment for gout^b^	Urate-lowering medication adherence >80 %^c^	Likelihood of diet change with score of >8 (range, 0–10)^d^
Gender	*P* = 0.58	*P* = 0.03	*P* = 0.32	*P* = 0.41
Male	1.13 (0.74, 1.71)	0.61 (0.39, 0.95)^a^	0.68 (0.31, 1.47)	0.84 (0.55, 1.28)
Female	Ref	Ref	Ref	Ref
Age groups	*P* = 0.87	*P* = 0.16	*P* = 0.71	*P* = 0.023
21–40 years	1.39 (0.40, 4.71)	0.76 (0.22, 2.67)	0.81 (0.07, 9.05)	2.59 (0.51, 13.06)
41–60 years	1.14 (0.37, 3.53)	1.02 (0.32, 3.27)	1.01 (0.11, 9.26)	2.91 (0.63,13.58)
61–80 years	1.10 (0.34, 3.35)	0.64 (0.20, 2.08)	1.43 (0.16, 13.12)	4.76 (1.01, 22.37)*
>80 years	Ref	Ref	Ref	Ref
Race	*P* = 0.99	*P* = 0.60	*P* = 0.77	*P* = 0.48
African American	1.10 (0.41, 2.80)	0.91 (0.45, 1.85)	0.49 (0.11, 2.24)	1.35 (0.68, 2.70)
Hispanic or Latino	1.04 (0.33, 3.24)	0.92 (0.31, 2.77)	0.86 (0.09, 7.94)	1.13 (0.36, 3.54)
Asian	0.78 (0.19, 3.25)	0.59 (0.31, 1.12)	2.04 (0.76, 5.47)	0.63 (0.31, 1.29)
American Indian or Alaskan native	0.95 (0.31,2.89)	---	---	3.60 (0.31, 41.22)
Hawaiian or Pacific Islander	-----	4.15 (0.49, 35.11)	1.19 (0.13, 11.28)	2.23 (0.58, 8.52)
Other	0.96 (0.19, 4.83)	0.90 (0.34, 2.35)	1.60 (0.29, 8.76)	1.46 (0.56, 3.82)
White/Caucasian	Ref	Ref	Ref	Ref

*p-value <0.05

---, no odds ratio calculable since no patients in this category

^a^Reference category is ULT was not prescribed

^b^Reference category is preference for non-pharmacological treatment options (cherry extract or gout-specific diet modification) for gout

^c^Reference category is urate-lowering medication adherence of ≤80 % in the last month

^d^Reference category is the likelihood of diet change with a score ≤8 (range 0–10)

**ULT adherence**: Of those who were prescribed ULT, 251/285 (88.1 %) were taking ULT, 20/285 (7 %) quit taking ULT, 2/285 (0.4 %) were taking ULT only when they had a gout flare and 13/285 (4.5 %) did not respond to this question. Of those taking ULT, 148/251 (59 %) reported that they never missed ULT medication in the past 30 days (100 % adherence), 49/251 (19.5 %) missed it 1–5 days, 26/251 (10.3 %) missed ULT 6–14 days and 28/251 (11.2 %) missed it >15 of the past 30 days (Table 1). Thus, 197/251 (78.5 %) respondents reported ULT adherence >80 % (Appendix 1). In univariate (Appendix 2) and multivariable-adjusted analyses (Table 2), gender, race, and age were not significant predictors of ULT adherence >80 %.

**Patient preference for best gout management strategy**: Of survey respondents, 56 % preferred ULT as a lifelong treatment for gout, 24 % reported that they preferred cherry extract, 16 % preferred diet modification and 4 % preferred none of the treatments (Table 1). Unadjusted rates of treatment choices are shown in Appendix 2 and Appendix 3. Treatment choices (ULT vs. diet modification vs. cherry extract vs. none) did not differ significantly by age or gender, but differed by race in unadjusted analyses (*p* = 0.011; Appendix 3). In multivariable-adjusted analyses, men had significantly lower odds of preferring ULT as the lifelong treatment choice for gout vs. others (diet modification or cherry extract) (*p* = 0.03; Table 2).

**Patient motivation for lifelong dietary modification as gout management strategy**: Of survey respondents, 38.3 % were highly motivated to make a lifelong dietary modification to improve their gout (score of 9–10 on 0–10 likelihood scale) (Appendix 4). In unadjusted analyses, age was significantly associated with higher level of willingness to modify diet (*p* = 0.02; Appendix 2), confirmed in multivariable-adjusted analyses (*p* = 0.02; Table 2). Specifically, compared to >80 years, adults 61–80 years old were significantly more likely to make lifelong diet change with an odds ratio of 4.76 (95 % CI 1.01, 22.57; *p* = 0.02 for age; Table 2).

**Sensitivity Analyses**: These were performed to assess whether patient preference for non-pharmacological treatments impacts the odds of prescription of ULT for gout or ULT adherence >80 %. In multivariable-adjusted models, patient preference for non-pharmacological therapy over pharmacological therapy was not associated with either receipt of ULT prescription or with ULT adherence >80 %, respective odds ratios were 1.00 (95 % CI, 0.69, 1.46; *p*-value = 1.00) and 1.02 (95 % CI, 0.51, 2.04; *p*-value = 0.96) (Appendix 5).

## Discussion

Our study focused on pharmacological and non-pharmacological strategies for the management of gout. Our study sample is representative of gout patients, and the demographic characteristics of our survey respondents were similar to patients in previous studies of gout [13, 24, 25], although all were visitors to a gout website with no control over which viewers decided to respond to this short, targeted questionnaire. Rates of ULT use are similar to previously published data [12].

We found that none of the patient characteristics studied, i.e., gender, race, and age, were significantly associated with the likelihood of either receiving a ULT prescription or ULT adherence >0.80. These findings add new knowledge to this area of research. Our results agree with a previous database study that found no sex difference in unadjusted rates of allopurinol prescription [25], despite differences in sampling method (internet survey vs. health maintenance organization [HMO] database). Our findings of no association of age or gender with ULT adherence are similar to that reported for a study of HMO data [13], but are somewhat different from another study that reported that female gender and older age are associated with higher allopurinol adherence [26]. We made several novel and interesting observations in this study that merit further discussion.

Our study shows that a relatively large proportion of the gout population, i.e. 40 %, reported that they preferred non-pharmacological interventions for gout (cherry extract by 24 % and diet modification by 16 %) rather than ULT for long-term gout management. This is an interesting finding as these patients were the viewers of a gout website that emphasizes the importance of ULT as the key pharmacologic therapy for gout. These findings are at least partly in line with a previous finding that 50 % Americans use various dietary supplements including vitamins, minerals, herbs, extracts/concentrates etc. as treatments or to stay healthy [27]. Our recent gout Internet survey study showed that 50 % of gout patients reported the use of dietary supplements or natural treatments, most commonly cherry extract or juice, but also vitamins, celery seed, turmeric, lemon juice etc., for gout [12]. However, the current survey was worded to ask about reliance primarily on these as a treatment rather than supplementary use.

An interesting finding in our study was that patient preference for non-pharmacological treatments was not associated with either the receipt of ULT prescription or ULT adherence >80 %. This finding should reassure health care providers that patients preferring non-pharmacological treatments are not any less likely than those preferring pharmacological treatment to start ULT or adhere well to ULT. It is possible that discussing these non-pharmacological options during a clinic visit in a patient-centric approach can lead to setting of common goals by patient-physician team that may lead to higher patient satisfaction and possibly more success in achieving treatment goals.

Evidence has emerged in the last decade showing the potential contributions of dietary supplements such as cherry extract [10, 28] and diet modification [9, 23] to the management of gout; both are included as adjunctive strategies in the ACR and EULAR gout treatment guidelines [7, 8]. A recent Cochrane review found that only two clinical trials that evaluated the efficacy of dietary supplements in gout have been published to date [29]. There is a general agreement to encourage diet modification for the management of gout [7, 30]. The role of supplements (cherry extract, available as capsules/tablets or cherry juice, available as concentrate) in the management of gout is an area of active research [10, 28]. Rather, specific diet components may be responsible for triggering flares in someone with established gout [31, 32].

To our knowledge, this is one of first large studies to describe patient preferences for treatment. A large proportion of gout patients preferred non-pharmacological options to ULT. This finding will require further clarification to understand and perhaps guide patient preferences for gout treatment. We assessed patient preference for the most preferred treatment strategy, not their current use, which might include a combination of these strategies, at least in some patients. This additional information may have provided more insight about concordance between patient preference and behavior.

Diet plays an important role in management of gout [9]. More than one-third of the study respondents with gout had very high motivation (score, 9–10) to make a lifelong dietary change to improve their gout management and outcomes. More than 50 % rheumatologists do not refer their gout patients to dietary services for dietary advice, indicating a difference in perceived effectiveness of dietary management in gout between rheumatologists who recognize the significant but limited effect of diet change on serum urate levels [33] vs. the patients and nutrition professionals who likely rank it higher and more useful. Also, a large proportion of gout patients do not follow evidence-based diet modification for gout management [33]. Thus, barriers and challenges exist regarding the need for and implementation of diet modification as an adjunctive or main strategy (for milder cases) for gout management.

Given high patient interest and few available data, we believe this area needs more research. There is a need for clinical trials that assess the comparative efficacy/effectiveness and harms of these non-pharmacological treatment options for gout. To our knowledge, no such trials have yet been performed. Such trials were rated as the highest research priority by patients [18]. Patients prefer and commonly use complementary medicines and dietary modifications for gout management [15, 34]. One previous study showed that the use of complementary medicines may be a barrier to high ULT adherence [15], but our study found no evidence to support this notion. This indicates that more research is needed to assess the impact of use of non-pharmacological treatments on pharmacologic treatments for gout.

A recent study found that patient-centered interventions that focus on education about pharmacologic therapy and lifestyle modifications result in a greater proportion of patients achieving recommended serum urate levels [35, 36]. A comprehensive clinical approach combining non-pharmacological and pharmacological interventions for gout should be evaluated to ascertain if it increases patient motivation and improves ULT adherence [9]. Novel approaches need to be tested to discover the most optimal approach that can benefit the largest proportion of patients with gout.

A key limitation of our study was patient self-selection to an optional Internet survey that clearly focused on diet modification and cherry extract supplement. Response rate could not be assessed (beyond 499 of the 525 who clicked on the survey) since the survey was only for patients with physician-diagnosed gout and was optional (not mandatory), and there was no way to assess as to what proportion of visitors to the website have physician-diagnosed gout (or even self-reported) gout. However, during the study period, the website recorded 89,268 unique visits. The study findings may also not be generalizable to all gout patients, but only to gout patients who use the Internet. In addition, gout patients using the Internet are more likely to respond to the survey due to an overwhelming exposure to websites devoted to promoting cherry juice or other dietary supplements. Also, the validity of self-report of physician diagnosis of gout may be questioned.

Limited resources prevented us from obtaining further confirmation of gout diagnosis. A cross-sectional study design does not allow for establishing causation, i.e., the effect may be in either direction for an association. The validity of self-reported ULT use is unknown. We are not sure whether self-reported ULT was more or less accurate than a traditional approaches to assess adherence, such as pharmacy-based prescription fill data or pill counts. Social desirability could have led to a less accurate reporting vs. the anonymity of the survey that could have led to a more accurate reporting of medication adherence. The pharmacy-based approach has its own limitations. We did not collect data on the duration of gout, which might influence the likelihood of use of dietary supplements, since the focus of the survey was about patient preference.

The main strengths of our study were a focus on non-pharmacological options for the management of gout, which are of interest to patients [16, 18, 37]. A large diverse sample that included a large proportion of minorities and females makes our study findings more generalizable than those from the previous studies, which had less diverse samples.

## Conclusions

In conclusion, this study showed that only 57 % of gout patients reported being prescribed ULT by their healthcare providers. Forty percent of gout patients responding to the survey stated that they preferred non-pharmacological interventions such as cherry extract and diet modification to urate lowering drugs for gout management. We propose that there is a need for studies that can examine the role of non-pharmacological interventions in the management of gout. Researchers and funding agencies should take these priorities into consideration while setting future research agenda for gout and designing studies.

### IRB approval

The University of Alabama at Birmingham’s Institutional Review Board approved this study and all investigations were conducted in conformity with ethical principles of research.

## Appendix 1

**Table 3:** ULT adherence rates by gender, race and age^a^

	ULT adherence ≤50 % *N* = 28 (11.2 %)	ULT adherence 51–80 % *N* = 26 (10.3 %)	ULT adherence >80 % *N* = 195 (78.5 %)	*P*-value
Gender				0.40
Male	11.7	11.7	76.6	
Female	9.5	6.3	84.1	
Race				0.49
White/Caucasian	10.6	9.0	80.3	
African American	9.5	4.8	85.7	
Hispanic or Latino	0.0	16.7	83.3	
Asian	22.7	18.2	59.1	
American Indian or Alaskan Native	0.0	0.0	100.0	
Hawaiian or Pacific Islander	0.0	20.0	80.0	
Other	14.3	14.3	71.4	
Age groups				0.63
21 to 40	12.9	03.2	83.9	
41 to 60	9.0	11.5	79.5	
61 to 80	14.3	11.0	74.7	
>80	0.0	14.3	85.7	

^a^all values are in percentages

## Appendix 2

**Table 4:** Univariate odds of treatment choice, ULT adherence and likelihood diet change for gout by gender, race and age

Patient characteristics	Patient preference for choice of ULT as the treatment for gout^a^	Urate-lowering medication adherence >80 %^b^	Likelihood of diet change^c^
Gender	*P* = **0.04**	*P* = 0.21	*P* = 0.15
Male	**0.64 (0.42, 0.98)***	0.62 (0.29, 1.31)	0.74 (0.50, 1.12)
Female	Ref	Ref	Ref
Age groups	*P* = 0.32	*P* = 0.78	*P* = **0.02**
21–40	0.83 (0.24, 2.87)	0.89 (0.84, 9.44)	2.89 (0.58, 14.25)
41–60	1.01 (0.32, 3.20)	1.32 (0.15, 11.52)	2.84 (0.61, 13.10)
61–80	0.69 (0.22, 2.21)	1.58 (0.18, 13.96)	**4.86 (1.05, 22.57)***
>80	Ref	Ref	
Race	*P* = 0.49	*P* = 0.66	*P* = 0.32
African American	0.86 (0.43, 1.72)	0.49 (0.11, 2.23)	1.50 (0.77, 2.94)
Hispanic or Latino	0.92 (0.31, 2.71)	0.94(0.11, 8.31)	0.93 (0.31, 2.82)
Asian	0.55 (0.29, 1.03)	2.20(0.83, 5.80)	0.61(0.30, 1.21)
American Indian or Alaskan native	-----	-----	3.34 (0.30, 37.15)
Hawaiian or Pacific Islander	4.14 (0.50, 34.7)	1.17 (0.13, 10.85)	2.09 (0.55, 7.90)
Other	0.86 (0.33, 2.24)	1.88(0.35, 10.10)	1.67 (0.65, 4.31)
White/Caucasian	Ref	Ref	Ref

*Odds ratio with *p*-value <0.05; bold represents either the variable or the variable category with *p*-value <0.05

^a^Reference category is preference for non-medications (cherry extract or gout-specific diet modification) as the treatment strategy for gout

^b^Reference category is urate-lowering medication adherence of >80 % in the last month compared to < =80 %

^c^Likelihood of die change with score of 8 or more (score range 0–10; reference category <8)

## Appendix 3

**Table 5:** Treatment choice by gender, race and age^a^

Patient characteristics	Cherry extract *N* = 122 (24.4 %)	Diet modification *N* = 79 (15.8 %)	Gout medication *N* = 277 (55.5 %)	None *N* = 21 (4.25 %)	*P*-value
Gender					
Male	25.5	17.7	53.3	3.5	0.091
Female	21.4	10.7	61.8	6.1	
Race					**0.011**
White/Caucasian	24	15.1	56.6	4.3	
African American	28.9	13.2	52.6	5.3	
Hispanic or Latino	14.3	28.6	57.1	0	
Asian	26.7	28.9	44.4	0	
American Indian or Alaskan Native	0	0	66.7	33.3	
Hawaiian or Pacific Islander	0	11.1	66.7	22.2	
Other	44.4	0	55.6	0	
Age groups					0.30
21 to 40	29.5	9.8	52.5	8.2	
41 to 60	20.9	16	59.8	3.3	
61 to 80	27.9	17.3	50.3	4.5	
>80	15.4	23.1	61.5	0	

^a^all values are in percentages

Bold represents *p*-value <0.05

## Appendix 4

**Table 6:** Univariate association of the likelihood of diet change (scale 0–10) by gender, race and age^a^

Patient characteristics	Likelihood of life-long diet modification 0 to 8 scale *N* = 308 (61.7 %)	Likelihood of life-long diet modification 9–10 scale *N* = 191 (38.3 %)	*P* value
Gender			0.15
Male	63.6	36.4	
Female	56.5	43.5	
Race			0.30
White/Caucasian	62.5	37.5	
African American	52.6	47.4	
Hispanic or Latino	64.3	35.7	
Asian	73.3	26.7	
American Indian or Alaskan Native	33.3	66.7	
Hawaiian or Pacific Islander	44.4	55.6	
Other	50	50	
Age groups			**0.01**
21 to 40	65.6	34.4	
41 to 60	66	34	
61 to 80	53.1	46.9	
>80	84.6	15.4	

^a^all values are in percentages. Bold represents *p*-value <0.05

## Appendix 5

**Table 7:** Sensitivity analyses for prescription of ULT and for ULT adherence >80 %, additionally adjusting for patient preference for pharmacological vs. non-pharmacological treatments

Patient characteristics	ULT prescribed^a^	Urate-lowering medication adherence >80 %^b^
Gender	*P* = 0.58	*P* = 0.12
Male	0.90 (0.58, 1.38)	2.10 (0.81, 5.44)
Female	Ref	Ref
Age groups	*P* = 0.98	*P* = 0.91
21–40 years	0.82 (0.24, 2.82)	0.89 (0.08, 10.2)
41–60 years	0.88 (0.28, 2.71)	1.00 (0.11, 9.31)
61–80 years	0.92 (0.29, 2.91)	1.27 (0.13, 12.00)
>80 years	Ref	Ref
Race	*P* = 0.99	*P* = 0.64
African American	1.01 (0.50, 2.04)	0.25 (0.03, 1.97)
Hispanic or Latino	1.37 (0.46, 4.05)	0.85 (0.09, 7.83)
Asian	1.12 (0.59, 2.12)	1.96 (0.72, 5.31)
American Indian or Alaskan native	-----	---
Hawaiian or Pacific Islander	1.84 (0.40, 8.42)	1.96 (0.17, 23.00)
Other	0.90 (0.58, 2.78)	1.61 (0.29, 8.86)
White/Caucasian	Ref	Ref
Choice of preferred therapy for gout		
Non-ULT	1.00 (0.69, 1.46)**	1.02 (0.51, 2.04)***
ULT	Ref	Ref

***p*-value =1.00; ****p*-value =0.96

---, no odds ratio calculable since no patients in this category

^a^Reference category is ULT was not prescribed

^b^Reference category is urate-lowering medication adherence of ≤80 % in the last month

## Abbreviations

ACR: American College of Rheumatology
CI: confidence interval
EULAR: the European League Against Rheumatism
ULT: urate-lowering therapy
